# Rehearsal development as development of iterative recall processes

**DOI:** 10.3389/fpsyg.2015.00308

**Published:** 2015-03-30

**Authors:** Martin Lehmann

**Affiliations:** German Institute for International Educational Research, Center for Education and DevelopmentFrankfurt, Germany

**Keywords:** rehearsal, free recall, episodic memory, grouping, strategy use, cognitive development, serial position curve

## Abstract

Although much is known about the critical importance of active verbal rehearsal for successful recall, knowledge about the mechanisms of rehearsal and their respective development in children is very limited. To be able to rehearse several items together, these items have to be available, or, if presented and rehearsed previously, retrieved from memory. Therefore, joint rehearsal of several items may itself be considered recall. Accordingly, by analyzing free recall, one cannot only gain insight into how recall and rehearsal unfold, but also into how principles that govern children’s recall govern children’s rehearsal. Over a period of three and a half years (beginning at grade 3) 54 children were longitudinally assessed seven times on several overt rehearsal free recall trials. A first set of analyses on recall revealed significant age-related increases in the primacy effect and an age-invariant recency effect. In the middle portion of the list, wave-shaped recall characteristics emerged and increased with age, indicating grouping of the list into subsequences. In a second set of analyses, overt rehearsal behavior was decomposed into distinct rehearsal sets. Analyses of these sets revealed that the distribution of rehearsals within each set resembled the serial position curves with one- or two-item primacy and recency effects and wave-shaped rehearsal patterns in between. In addition, rehearsal behavior throughout the list was characterized by a decreasing tendency to begin rehearsal sets with the first list item. This result parallels the phenomenon of beginning recall with the first item on short lists and with the last item on longer lists.

## Introduction

When children are asked to remember a list of words, active rehearsal of several items together contributes greatly to their recall performance. With increasing age, children increasingly employ active rehearsal and, as a consequence, profit from this behavior. For joint rehearsal of several items, however, previously presented items have to be recalled and thereby made available for rehearsal. As a consequence, rehearsal during input is more than mere parrotry of items and seems to be the basis and the consequence of successful recall behavior. The analyses in the present study were motivated by the assumption that both rehearsal and recall are based on similar cognitive processes. For this purpose, data from a longitudinal study on children’s rehearsal behavior during overt rehearsal and subsequent free recall (Lehmann and Hasselhorn, 2007, 2010) were reanalyzed. Additionally, the data from three assessments that had not yet been published were included in the current analyses. Lehmann and Hasselhorn (2007, 2010) had substantiated the close relationship between rehearsal behavior and recall in children between 8 and 10 years of age. Their first major finding (Lehmann and Hasselhorn, 2007) pointed out that rehearsal development was characterized by a gradual change from passive behavior (labeling) to active behavior (cumulative rehearsal). Further, the more extensively children rehearsed actively during the presentation of a list, the better their recall was. Their second major finding (Lehmann and Hasselhorn, 2010) concerned the link between rehearsal dynamics and recall dynamics: items recalled in succession came predominantly from nearby serial positions and were particularly frequently rehearsed together (cumulatively). This effect turned out to increase with age. These findings on extended active rehearsal behavior during presentation of a list and on the close resemblance between rehearsal content and recall dynamics provide a good starting point for emphasizing the relationship between rehearsal and recall.

It seems unquestionable that rehearsal is critical for successful recall. Knowledge about its mechanism, however, is less well established. Starting with the seminal work by Ornstein and colleagues in the 1970s (for an overview, see Ornstein and Naus, 1978), studies on rehearsal development demonstrated that rehearsal behavior is closely linked with free recall performance and that this behavior changes with age. A method that permits the direct observation of children’s rehearsal techniques is the overt rehearsal procedure (Rundus and Atkinson, 1970; Rundus, 1971). Here, participants are required to say out loud any words that come to mind while items are presented. When observing older and younger children in that manner, it became obvious that older and younger children’s rehearsal style differed. Whereas younger children (under about 8 years of age) tended to rehearse each newly presented item only once or in minimal combination with other items (e.g., Cuvo, 1975; Ornstein et al., 1975), older children intermixed various items in a rehearsal set. A rehearsal set is defined by the interval between the presentation of consecutive items in a list and its size is defined by the number of different items included in that set. As such, rehearsal set sizes increased over the first serial positions and leveled off at asymptotes that differed with respect to children’s rehearsal activity and accordingly to children’s age.

As noted above, these findings were confirmed and extended in a longitudinal study with children from 8 to 10 years of age (Lehmann and Hasselhorn, 2007). Lehmann and Hasselhorn (2007) identified in their analyses different passive (labeling, single-word rehearsal) and active (cumulative rehearsal) rehearsal strategies. Interestingly, at any age, children used a combination of active and passive rehearsal behavior when trying to remember the items. When children were younger, they were able to rehearse actively at the beginning of the list but rapidly ceased to do so in the course of the list and tended to repeat only the currently presented item thereafter. When they were older, children again rehearsed actively at the beginning of the list but were better able to continue to do so throughout the list and were passively rehearsing only at late list portions. Taken together, the findings from studies on spontaneous rehearsal behavior in children suggest that when children are older, they increasingly redintegrate previously presented items in new rehearsal sets and they increasingly do so deliberately throughout the list.

In addition to the changes in rehearsal style, studies found age-related differences in children’s recall performance. These differences were most prominent in the primacy and prerecency section of the list. Accordingly, it was assumed that the increase in active rehearsal would be responsible for the increase of recall from the primacy section of the list. From the perspective of multistore models (e.g., Atkinson and Shiffrin, 1968; Raaijmakers and Shiffrin, 1981), recall of items from the initial and middle serial positions might be considered recall from a long-term store (LTS), whereas recall of items from the recency portion of the list might be considered recall from a short-term store (STS). According to this framework, rehearsal development from passive to active rehearsal style would affect recall from LTS. LTS keeps associative information between items and between items and their context. By jointly memorizing several items together in different rehearsal sets, active rehearsal should create and strengthen the associative links between the items and transfer this information into LTS (Cuvo, 1975; Ornstein et al., 1977). In immediate free recall, the recency effect seems to be fairly unaffected by rehearsal development and therefore by age. The recency effect is considered to be a consequence of participants first recalling items that are kept in the STS. At the end of the list, items in the STS are assumed to come mainly from the recency portion of the list.

Another perspective on the consequences of rehearsal for free recall is given by approaches that take into account the retention interval between an item’s last instantiation and recall (e.g., Tan and Ward, 2000). On the one hand, if no additional rehearsal takes place due to children’s passive rehearsal style or under conditions in which rehearsal is prevented, the retention interval of the early items will increase and in turn recall of these items will decrease. On the other hand, when active rehearsal can take place, items will supposedly receive additional rehearsals, leading to more recent instantiations of the items and reducing thereby the retention interval. From this perspective, free recall of items is recency based because it is related to the last instantiations of the items. For instance, when considering the number of rehearsals items from different serial positions receive, it has been demonstrated that items from the beginning of the list are far more frequently repeated in later rehearsal sets than items from the middle or recency portion of the list (e.g., Rundus, 1971) and that this tendency increases with age (Ornstein et al., 1975). Consequently, children’s rehearsal behavior reassembles the to-be-learned material for later recall. Tan and Ward (2000) demonstrated in adults that recall performance in free recall was determined by the number, distribution, and recency of items and rehearsals. In their recency-based account of free recall, they emphasized that the traditional U-shaped serial position curves, when replotted according to when items were last rehearsed, displayed extended recency effects with no primacy. These so-called functional serial position curves therefore reveal participants’ selective rehearsal for primacy items. Items that are not, or only little, rehearsed (i.e., not redintegrated in later rehearsal sets) have therefore only low probabilities to be recalled.

A study by Stone (unpublished master’s thesis, as cited in Ornstein and Naus, 1978) supports the view that recall characteristics change in favor of elevated prerecency recall when early presented items are kept available for repeated rehearsal. In this study, second and sixth graders were instructed to actively rehearse a list of items, either while the so far presented items remained visible for rehearsal or while they did not remain visible for rehearsal. In the visibility condition, items were not removed from the children’s sight after their presentation but were placed in front of them until the recall signal was administered. Stone found that second graders were more actively rehearsing when they were provided with the items available for rehearsal and performed approximately at the level of sixth-grade children who did not have the previously presented items available. In addition, the item availability manipulation altered the shape of the serial position function and resulted in less differentiation between the beginning and middle sections of the curve (see also, Ornstein et al., 1985). Hence, the longer children are able to actively rehearse previously presented items and to keep them in mind, the more often and the more recently these items are represented in a mnemonic record.

When analyzing single rehearsal sets, Tan and Ward (2000) found that the distribution of rehearsals paralleled serial position curves in immediate serial recall. More precisely, at later rehearsal sets these serial position curves were characterized by extended primacy and recency. In light of this finding, it is not surprising that processes that generate recall should be very similar to those that generate rehearsal (Tan and Ward, 2000; Laming, 2008): each new compilation of a rehearsal set is based on the retrieval of previously presented and previously rehearsed items. Accordingly, the probability of redintegration of previously presented items for rehearsal seems to be subjected to mechanisms that are similar to those for recall.

The notion that rehearsal at each specific moment during the study phase is based on previously presented and rehearsed items and that this rehearsal in turn lays the foundation for subsequent rehearsal is further supported by Laming (2006, 2008). Laming developed an algorithm by which he was able to predict sequences of recall on the basis of prior sequences of stimuli and rehearsals in adults. He assumed that item presentations and rehearsals of the items form an internal sequence. When an item is retrieved for rehearsal (or recall) it is written to the head of that sequence. The accessibility of the items is assumed to decrease with increasing distance from the current point in the sequence. During the formation of the mnemonic record, preceding subsequences of previous rehearsals are retrieved for rehearsal. As such, the mnemonic record for so far presented and rehearsed sequences of items is the product of a cumulative process of the repetition of previous runs of rehearsals and consequently the basis for the extension of the sequence. Early in the list, there are only the very few thus far presented items available. Accordingly, the first sequences are constituted by consecutive presentations and rehearsals from these early list items (Laming, 2010). The further the item presentations within the list proceed, the higher becomes the probability to fail to recall previously presented items resulting in selectively compiled rehearsal sets. In turn, rehearsal at each rehearsal opportunity in the list equals to the ability and the failure to recall previously presented and rehearsed information. Accordingly, patterns of rehearsal are assumed to correspond to patterns and regularities of free recall.

As mentioned earlier, little is known about the mechanism of rehearsal-recall processes in children and its respective development. The motivation behind the present analyses was twofold. The first main purpose was to identify whether children’s free recall behavior was recency based. More specifically, the goal of the analyses was to examine whether children were selectively rehearsing and whether such selective rehearsal, in terms of reassembling presented and previously rehearsed items, would result in corresponding recall characteristics. A recency-based account of free recall may potentially be very sensitive to age-related changes in rehearsal-recall correspondencies: with increasing age, children should be more and more able to integrate items from early in the list in later rehearsal sets. Consequently, with increasing age, these items should be less distant from the end of the list and therefore be better recalled. Recall of items from the end of the list should, however, not undergo age-related changes because recall of these items seems to be independent of children’s rehearsal.

The second main purpose of the present analyses was whether rehearsal followed the same mechanisms as free recall does and how rehearsal behavior unfolds at different rehearsal opportunities during item presentation. By analyzing the rehearsal and recall processes in free recall, one can examine how previous patterns of rehearsals and recalls influence subsequent rehearsal and recall patterns. If, as assumed above, recall and rehearsal are generated by the same process, rehearsal sets should reveal the same phenomena as free recall does over and over again throughout the list, above all, primacy and recency. Recency might arise due to retrieval from the STS loaded with items from the recency portion of the list or due to a short retention interval between the last instantiation of an item and the current rehearsal opportunity. Primacy might arise due to the tendency of early list items to be more often rehearsed than items later on the list. Due to increasing ability to actively rehearse, I anticipate age-related changes in these phenomena with increasing primacy emerging when children grow older.

Another phenomenon is participants’ tendency to start free recall with either items from the primacy or from the recency portion of the list. The so-called probability of first recall (PFR) gives information about the starting point of a rehearsal sequence and accordingly of the mnemonic record. Grenfell-Essam et al. (2013; see also, Ward et al., 2010) found that adult participants tended to initiate recall with the first list item when lists were short, but that they tended to initiate free recall with one of the last list items when list length increased. As such, free recall (and correspondingly rehearsal) of short lists (few items) seems fairly primacy based whereas free recall (and again correspondingly rehearsal) of long lists (many items) seems fairly recency based. When rehearsal and recall are equated, I expect that rehearsal behavior early in the list (i.e., with only few items presented) closely resembles recall behavior of short lists. Thus, there should be a stronger tendency to initiate a rehearsal set with a primacy item. Accordingly, I expect rehearsal behavior later in the list to resemble recall of longer lists resulting in a tendency to initiate a rehearsal set with a recency item. In addition, due to the age-related increase in active rehearsal behavior, the tendency to initiate rehearsal with a primacy item should be stronger when children are older.

## Materials and Methods

### Participants and Basic Longitudinal Design

Fifty-four children participated in all assessments of the longitudinal study over the course of four and a half-years. The children were recruited from 16 elementary schools in the area of Göttingen, Germany, and came primarily from middle-income families. When they were assessed for the first time, children were in grade 2 (*M* age = 8,3 years, *SD* = 6 months). Following their first assessment, children were tested again every 6 months for a total of nine times. At the ninth and last measurement point, children thus were in grade 6 (*M* age = 12,2 years, *SD* = 6 months). The task in question, the immediate free recall task, was applied to all children at assessments 1–6 and 8–9, respectively. The analyses presented here include data from seven time points in the longitudinal study. The reason for omitting the first measurement point from the current analyses is that material and amount of to-be remembered information in the task differed between the first and all following measurement points. The present analyses include three assessments (6, 8, and 9) that were not reported in previous analyses. For better legibility, the assessments used in the presented analyses were renumbered from “1” to “7,” instead of being identified by their original number.

### Materials and Procedure

At each assessment, children were presented with three immediate free recall trials, each consisting of a list of 12 unrelated words. List items were administered simultaneously acoustically (from CD) and visually (on 5 cm × 5 cm picture cards) at a rate of one item every 8 s. Each card was visible on a desk in front of the child for 8 s, after which the next item was acoustically presented and the card replaced by the next picture card. Children were instructed to “think aloud,” that is to verbalize what they were thinking or doing while memorizing the series of items (for a similar procedure, see Guttentag et al., 1987). After the presentation of the entire list, children were prompted to recall as many items as possible in any order they wished. The recall phase ended after a 1-min interval in which the child was not able to recall any more words. In order to ensure that children were able to meet task requirements and to familiarize them with the thinking-aloud procedure, practice trials of six items were used. Children were tested individually and both their learning and recall behavior were videotaped.

### Coding and Scoring

Children’s verbalizations during the study phase were assessed, coded, and scored at each rehearsal opportunity, that is, at each 8 s interval between two consecutive item presentations. Three independent raters agreed on about 92% of the verbalizations. The few disagreements were resolved through reinspection of the recordings. For scoring and coding of recall behavior, again audio and video recordings were used. Three independent raters agreed on about 95% regarding recall order and amount. Again, disagreements were resolved through reinspection of the recordings.

## Results

In accordance with previous research (Lehmann and Hasselhorn, 2007, 2010), two standard analyses for qualitative and quantitative development in verbal rehearsal were performed. Firstly, development of the quality of rehearsal assessed by the rehearsal set size was investigated. The rehearsal set size is the number of different items studied within an interstimulus interval and thus gives information about children’s strategic behavior: a rehearsal set size of 1 indicates that children either repeat a single item only once (labeling) or several times (single word rehearsal) and a rehearsal set size of two or more items indicates that children repeat several items together (cumulative rehearsal). In the longitudinal study, rehearsal set size steadily increased from the first to the penultimate assessment and slightly dropped thereafter. Respective mean values for measurement points 1–7 were: 1.3 (*SD* = 0.8), 1.6 (*SD* = 1.1), 1.9 (*SD* = 1.2), 2.0 (*SD* = 1.3), 2.3 (*SD* = 1.3), 2.5 (*SD* = 1.4), and 2.3 (*SD* = 1.4). A one-way analysis of variance (ANOVA) with assessments as the within-subject factor revealed a significant main effect of assessment, *F*(1,53) = 14.59, *MS*_e_ = 0.62, *p* < 0.001. Bonferroni *post hoc* comparisons indicated significant increases in rehearsal set size from assessments one to two and two to three, as well as a significant increase in rehearsal set size from assessment four to assessment six. Thus, the results indicate that after assessment four (end of grade 4) quality of rehearsal behavior stabilized in terms of active, cumulative rehearsal behavior.

Analyses of qualitative rehearsal development have to be accompanied by analyses on how often a specific learning behavior is demonstrated by the children. Accordingly, each interstimulus interval was coded on the basis of a strategy perspective and five different strategies were identified: labeling, single word rehearsal, cumulative rehearsal, no overt strategic behavior, and association (that is, coupling the presented list word with any additional self-generated information). Consistent with the analysis on qualitative rehearsal behavior, the number of interstimulus intervals employed with cumulative rehearsal turned out to increase from assessment to assessment and to consolidate around assessment four and thereafter. The respective percentages of cumulative rehearsal usage for assessments one to seven were: 27% (*SD* = 32), 40% (*SD* = 37), 49% (*SD* = 41), 52% (*SD* = 42), 56% (*SD* = 40), 59% (*SD* = 40), and 55% (*SD* = 42). A two-way ANOVA with assessments (1–7) and strategic behavior (5 different strategies as indicated above) as within-subject factors revealed a significant main effect of strategic behavior, *F*(4,212) = 27.05, *MS*_e_ = 0.45, *p* < 0.001, which was qualified by a significant interaction between assessment and strategic behavior, *F*(24,1272) = 5.79, *MS*_e_ = 0.43, *p* < 0.001. Bonferroni *post hoc* comparisons indicated that cumulative rehearsal was more often used than any other strategy from assessment four onward. Before assessment four, however, it was equally often used as labeling. Labeling was more often used than single word rehearsal during assessments one and two but not thereafter and was more often used than association at all assessments. Finally, no overt strategic behavior turned out to occur more often than association at all assessments but just as often as labeling or single word rehearsal (all *p*s < 0.05, Bonferroni-corrected).

In sum, with increasing age, qualitative, and quantitative changes in rehearsal behavior are apparent: firstly, throughout the elementary school years, children increasingly compile larger rehearsal sets of different items and secondly, children increasingly do so over the course of the list. Their ability to integrate several items into a rehearsal set implies that previously presented items were recalled. The larger the rehearsal set, the greater the respective recall performance of previously presented items. Therefore, cognitive mechanisms that determine recall may be the same that determine the item’s instantiations in each newly compiled rehearsal set. As such, rehearsal behavior throughout a list may be considered an iterative recall process that results in the free recall phase.

To provide a better understanding of this process, five detailed analyses were conducted. The analyses corresponded to similar analyses previously done in adults (see, e.g., Tan and Ward, 2000; Ward et al., 2010) and focused on how free recall is directly or indirectly linked to rehearsal. The first analysis investigated the nominal serial position curves that describe the recall probability of the items according to their given position in the list (nominal order). The second analysis examined where in the list, children began their recall. The third analysis examined the recall probability of the items according to their position of their last rehearsal. That is, items were rank ordered according to their last verbalization by each child resulting in so-called functional serial position curves (functional order). The fourth analysis investigated the number of each item’s redintegration in later rehearsal sets. The final set of analyses investigated the nominal serial position curves and initial rehearsals of the rehearsal sets throughout the list. In other words, rehearsal sets were analyzed under the assumption that rehearsal and recall processes correspond.

### Nominal Serial Position Curves

**FIGURE 1:**
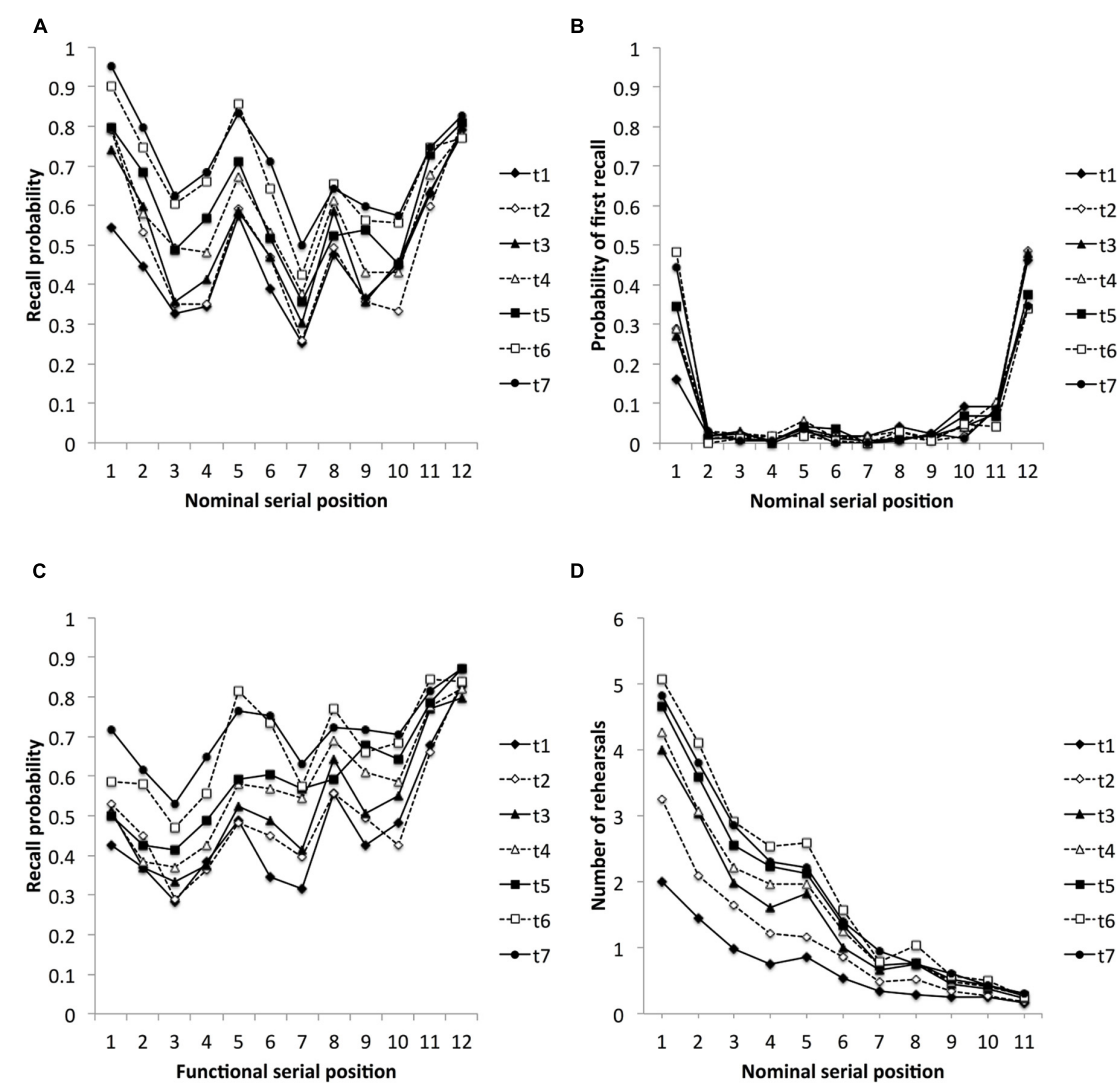
**Mean proportion of items recalled as a function of nominal serial position (A), probability of first recall (PFR) (B), mean proportion of items recalled as a function of functional serial position (C), and mean number of redintegrations in later rehearsal sets of items as a function of nominal serial position (D), for assessments (t) 1–7, respectively**.

**Figure 1A** displays the seven nominal serial position curves for assessments one to seven, respectively. Here, clear primacy and recency effects and two elevated peaks for items from serial positions 5 and 8 characterize the nominal serial position curves. To improve interpretability of the results in this section, serial positions were divided into groups of two successive items resulting in a within-subjects factor for serial positions with six levels (serial positions 1–2, 3–4, 5–6, 7–8, 9–10, and 11–12). A two-way repeated measures ANOVA with assessments (1–7) and serial positions (1–2, 3–4, 5–6, 7–8, 9–10, and 11–12) as within-subject factors revealed significant main effects for assessments, *F*(6,318) = 69.73, *MS*_e_ = 0.04, *p* < 0.001, and nominal serial positions, *F*(5,265) = 55.25, *MS*_e_ = 0.11, *p* < 0.001, and a significant Assessment × Nominal Serial Position interaction, *F*(30,1590) = 3.14, *MS*_e_ = 0.035, *p* < 0.001. Bonferroni *post hoc* comparisons revealed an age-related increase in recall performance at the primacy and the middle portion of the list: there were significant age-related increases in recall from serial positions 1–6 between assessments three and four and between assessments five and six, respectively. In addition, recall from serial positions 7–10 increased significantly between assessments five and six. There was no age-related increase at the recency portion of the list (all *p*s < 0.05, Bonferroni-corrected). Regarding recall performance between nominal serial positions, recall from serial positions 1–2 was inferior to recall from serial positions 11–12 only at the first assessment but not thereafter. Items from serial positions 1–2 were better recalled than items from all other serial positions at assessments two, three, five, six, and seven. Recall from serial positions 5–6 was better than recall from serial positions 3–4 at assessments one, two, three, six, and seven, and than recall from serial positions 7–8 at assessments three to seven. Finally, recall from serial positions 11–12 was better than that from serial positions 7–10 at all assessments (all *p*s < 0.05, Bonferroni-corrected). In sum, there was an age-related increase of the primacy effect and an age-invariant recency effect. In addition, there was an age-related increase of recall of items from the middle of the list.

Taking into account that children were required to maintain and recall items from supraspan lists (12 items), the observed peaks in the middle of the list at serial positions 5 and 8 suggest that children might have grouped the list into subsequences. Such grouping is very likely to produce primacy and recency effects within each subsequence. To test this interpretation, an additional set of *post hoc* analyses focusing on serial positions 5 and 8 was realized. Firstly, within each assessment Bonferroni corrected comparisons between serial position 5 and all other serial positions were conducted. These analyses revealed that recall from serial position 5 was better than that from most serial positions from the middle portion of the list: compared to serial positions 3, 4, 7, and 9 at all assessments, compared to serial position 6 at all but the third assessment, compared to serial position 10 at assessments four to seven, and compared to serial position 8 at assessments five to seven, all *t*s(53) > 3.12. Additionally, recall from serial position 5 was rarely smaller than recall from the primacy and recency portion of the list: compared to serial position 12 at assessments one to three, and compared to serial position 1 at the last assessment, all *t*s(53) > 3.12. Secondly, within each assessment Bonferroni corrected comparisons between serial position 8 and all other serial positions were conducted. Recall from serial position 8 was consistently larger when compared to serial position 7 (at assessments one to six). Compared to all other serial positions, an advantage was only sporadically detectable: compared to serial positions 3 and 4 at assessment three, compared to serial position 9 at assessments three and four, and compared to serial position 10 at assessments two and four, all *t*s(53) > 3.11. Furthermore, recall from serial position 8 was consistently smaller than that from serial position 1 (at assessments two, and four to seven) and than that from serial position 12 (at assessments one to five, and seven), all *t*s(53) > 3.14. Under the assumption that grouping of items into subsequences took place (with primacy and recency within each subsequence), the formation of a first subsequence turned out to be fairly manifest (especially at later assessments) and the formation of a second subsequence turned out to be rather ambiguous.

### Probability of First Recall

Whereas serial position curves give information about recall probabilities as a function of item’s serial position, the PFR reveals which of the recalled items is the first in the output sequence (see, e.g., Ward et al., 2010). **Figure 1B** shows the PFR as a function of serial position and reveals a strong tendency for children to initiate recall with either the first or the final list item. Especially the tendency to start with the first list item, however, seems to change with age. A two-way repeated measures ANOVA with assessments (1–7) and serial positions (1, 2–11, and 12) as within-subject factors revealed significant main effects for assessments, *F*(6,318) = 5.03, *MS*_e_ = 0.017, *p* < 0.001, and nominal serial positions, *F*(2,106) = 40.11, *MS*_e_ = 0.378, *p* < 0.001, and a significant Assessment × Serial Position interaction, *F*(12,636) = 5.67, *MS*_e_ = 0.068, *p* < 0.001. Bonferroni *post hoc* comparisons revealed that there were age-related increases for initial responses from the first and the middle serial positions. Initial response from the first serial position was more frequently observed at assessments five to seven compared to assessment one and at assessment six compared to assessments three and four, respectively. Initial response from the middle serial positions was more frequently observed at assessment six compared to assessments one and four. Comparison between the different serial positions revealed for all assessments a clear tendency for initial responses to come either from the first or last serial position when compared to the middle serial positions. At assessment one, recall initiated more frequently with items from the last compared to the first serial position. At assessments two and three, initial response came tendentially more often from the last compared to the first serial position (Bonferroni-corrected *p* = 0.086 and *p* = 0.067, respectively; all other *p*s < 0.05, Bonferroni-corrected). At the following assessments, initial responses had the same probability to come either from the first or last serial position.

### Functional Serial Position Curves

In this analysis, recall was replotted by functional order (see, e.g., Tan and Ward, 2000). Thus, serial position curves were reordered on the basis of each item’s last verbalization by the child. Accordingly, it was children’s rehearsal behavior that assigned each item a new (functional) serial position within the list. To identify each item’s new position, children’s coded verbalizations were stepped through, beginning by the last verbalization and continuing in reverse order. The first encounter of an item defined its functional position. If this item was found again later (namely earlier in the study phase) it was ignored. If, in the course of the study phase, a child had not named a specific item at all, its functional serial position was defined by the moment in the list, when it was presented. The number of such omissions was largest at assessment one (20%) but decreased thereafter (15%, at assessment two, and 9% at all subsequent assessments, respectively). Of the items that were not rehearsed overtly, at assessments 1–7, 34, 45, 39, 46, 52, 56, and 61% were recalled. To illustrate how functional serial position curves are generated, consider the following example of a child’s last verbalizations (presentations in bold): …, **7**, 7, 1, 2, **8**, 1, 2, 8, **9**, 1, 2, 8, **10**, 1, 2, 10, **11**, 1, 2, 10, **12**, 1, 2, 8, 12. When stepping backward through the verbalizations, the resulting rank ordered series of items is: …, 7, 9, 11, 10, 1, 2, 8, 12, and is assigned to serial positions …, 6, 7, 8, 9, 10, 11, 12. In a final step, recall of the respective serial positions is analyzed and recall probabilities are calculated (see also, Ward and Tan, 2004).

**Figure 1C** plots the recall probabilities for each functional serial position. A two-way repeated measures ANOVA with assessments (1–7) and serial positions (1–2, 3–4, 5–6, 7–8, 9–10, and 11–12) as within-subject factors revealed significant main effects for assessments, *F*(6,318) = 69.30, *MS*_e_ = 0.04, *p* < 0.001, and functional serial positions, *F*(5,265) = 57.74, *MS*_e_ = 0.10, *p* < 0.001, and a significant Assessment × Functional Serial Position interaction, *F*(30,1590) = 2.54, *MS*_e_ = 0.042, *p* < 0.001. Bonferroni *post hoc* comparisons revealed an age-invariant recency effect (functional serial positions 11–12) but an age-related increase in the primacy and middle portion of the functional serial position curves. More precisely, items that had an early rank ordered position (1–2) were better recalled at later assessments than at earlier assessments (assessment seven compared to assessments one to five, and assessment six compared to assessments one, three, and four, respectively). This effect proved significant again at later positions: functional serial positions 3–4 (better recall at assessment six compared to assessments one to three and at assessment seven compared to assessments one to five); functional serial positions 5–6 (better recall at assessments six and seven compared to assessments one to five and at assessments four and five compared to assessments one and two); functional serial positions 7–10 (better recall at assessments four to seven than at assessments one to three); all *p*s < 0.05, Bonferroni-corrected. Analyses regarding recall differences within the functional serial position curves revealed that items that were last rehearsed (functional serial positions 11–12) were better recalled than items from functional serial positions 1–4 and 7–10 at all assessments and than items from functional serial positions 5–6 at assessments one to five. Recall from functional serial positions 7–8 was better than recall from serial positions 3–4 at assessments two, three, five, and six. Recall from functional serial positions 5–6 was better than recall from functional serial positions 3–4 at assessments two to six, and than recall from functional serial positions 1–2 at assessment six (all *p*s < 0.05, Bonferroni-corrected). In sum, there was an age-invariant recency effect that was larger than recall from almost all earlier list portions, but from the middle list positions 5–6, at all assessments. In addition, recall of items that were dropped early increased toward the end of the longitudinal study.

### Number of Additional Rehearsals

**Figure 1D** shows the number of positions that an item from each individual nominal serial position had been moved further down the list (see, e.g., Tan and Ward, 2000). In other words, a small number implies that the item had received only little rehearsal behavior and was redintegrated in very few, if at all, additional rehearsal sets besides the one at which it had been presented. In contrast, a large number implies that an item from a specific serial position shows several instantiations throughout the study phase. Visual inspection of the functions in **Figure 1D** suggests a decrease of number of additional rehearsals as a function of the nominal serial position at all assessments. In addition, with increasing age, the amount of additional rehearsal increased. Because recency items (serial positions 11–12) had either one or no occasion to be redintegrated in later rehearsal sets, this list portion was omitted from the analyses. A two-way repeated measures ANOVA with assessments (1–7) and serial positions (1–2, 3–4, 5–6, 7–8, and 9–10) as within-subject factors revealed significant main effects for assessments, *F*(6,318) = 13.42, *MS*_e_ = 4.88, *p* < 0.001, and nominal serial positions, *F*(4,212) = 81.52, *MS*_e_ = 7.01, *p* < 0.001, and a significant Assessment × Nominal Serial Position interaction, *F*(24,1272) = 7.67, *MS*_e_ = 0.940, *p* < 0.001. Bonferroni *post hoc* comparisons revealed significant age-related increases in the number of redintegrations in later rehearsal sets: for serial positions 1–2 between assessments one and three and between assessments two and five; for serial positions 3–4 between assessments one and three, between assessments two and five, and between assessments three and six; for serial positions 5–6 between assessments one and three and between assessments two and five; for serial positions 7–8 between assessments one and three and between assessments two and six; for serial positions 9–10 between assessments one and three (all *p*s < 0.05, Bonferroni-corrected). In the course of the rehearsal process, items from the primacy portion of the list (serial positions 1–2) were more often redintegrated in later rehearsal sets than all subsequent items at all assessments, items from serial positions 3–4 were more often redintegrated than all subsequently presented items at assessments two to seven, items from serial positions 5–6 were more often integrated than all subsequently presented items at all assessments, and finally items from serial positions 7–8 were more often redintegrated than items from serial positions 9–10 at assessments two, six, and seven (all *p*s < 0.05, Bonferroni-corrected). In sum, there was a strong tendency in children’s rehearsal behavior to redintegrate items from the first portion of the list on later occasions compared to items from the latter portion of the list. This tendency was stronger at later assessments compared to earlier assessments.

### Rehearsal-Recall Similarities

The final set of analyses was based on the assumption that rehearsal processes during list learning rely on continuous recall. Ward (2002, p. 891) went so far as to suggest that “the words rehearsed at each RS [rehearsal set] may be considered to be mini–free recalls.” To recap, a rehearsal set is defined by the interval between the presentation of consecutive items. Accordingly, within these rehearsal sets (i.e., rehearsal intervals) patterns of rehearsal should reveal similar characteristics as does free recall. In the following sections, the distribution of rehearsals was analyzed within several distinct rehearsal sets throughout the list. More precisely, the probability of items rehearsed at rehearsal sets four, six, eight, and ten in the list were analyzed. Accordingly, the distribution of rehearsals within each of these rehearsal sets was considered to be the end-product of previous behavior analogous to the distribution of recalled items during the recall phase (see, e.g., Tan and Ward, 2000).

#### Nominal Serial Position Curves

**FIGURE 2:**
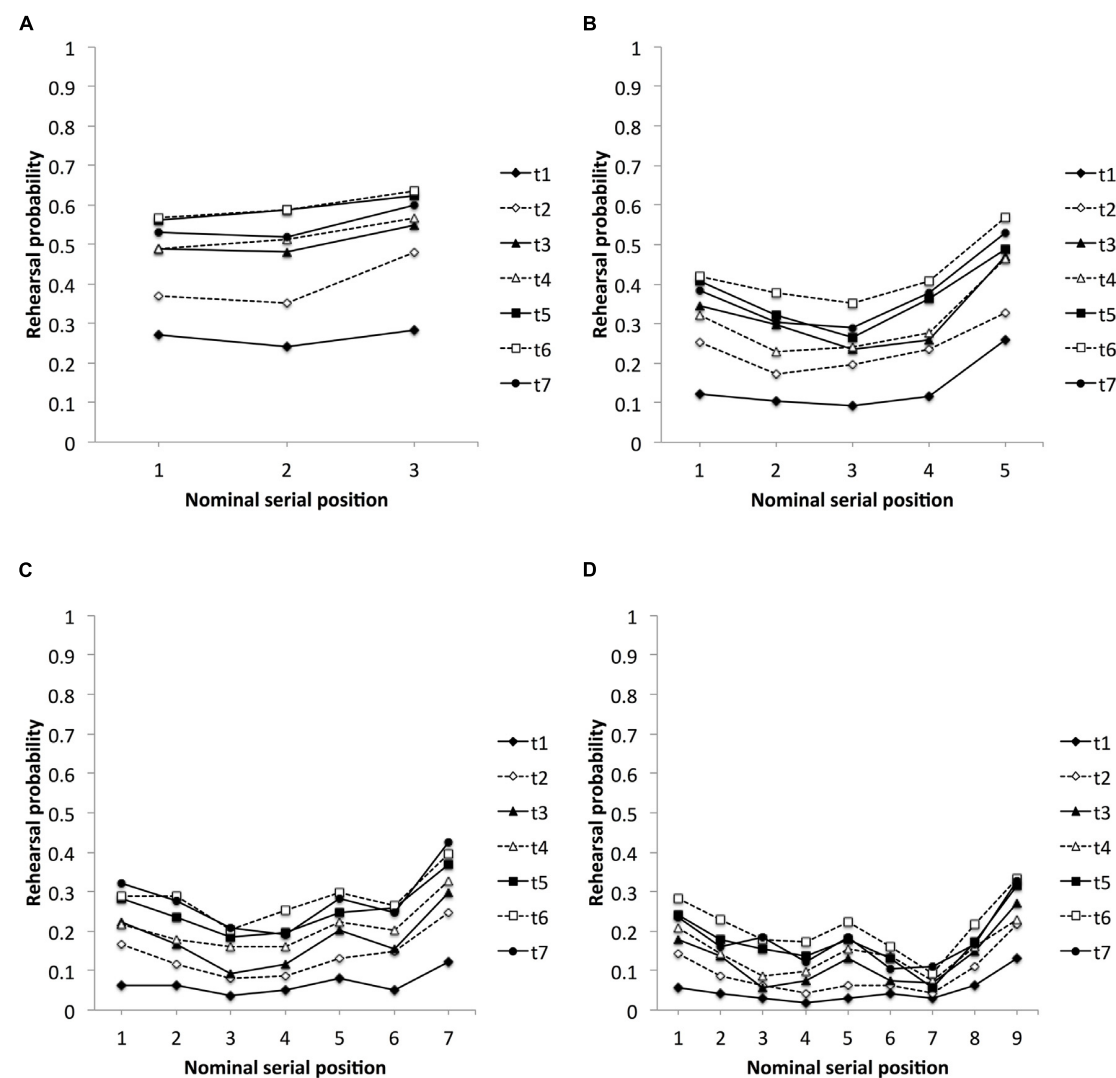
**Mean proportion of items rehearsed as a function of nominal serial position during rehearsal sets 4 **(A)**, 6 **(B)**, 8 **(C)**, and 10 **(D)**, for assessments (t) 1–7, respectively**.

**Figures 2A-D** plot rehearsal probabilities of previously presented items at four different rehearsal sets during the study phase. More precisely, it displays the rehearsal probability of items within rehearsal sets 4, 6, 8, and 10. The item that signaled the beginning of a new rehearsal set was excluded from the analyses (namely items 4, 6, 8, and 10 from the correspondent rehearsal sets). The reason for this procedure was to differentiate recall of previously presented items and mere repetition of the just presented item. As such, the analyses examined the serial position curves for: (i) the probability that items from serial positions 1–3 were rehearsed at rehearsal set 4, (ii) the probability that items from serial positions 1–5 were rehearsed at rehearsal set 6, (iii) the probability that items from serial positions 1–7 were rehearsed at rehearsal set 8, and (iv) the probability that items from serial positions 1–9 were rehearsed at rehearsal set 10. For each of the distributions of rehearsals at rehearsal sets 4, 6, 8, and 10, two-way repeated measures ANOVAs with assessments and serial positions as within-subject factors and rehearsal probability as the dependent measure were conducted.

The two-way repeated measures ANOVA with assessments (1–7) and serial positions (1–3) as within-subject factors revealed significant main effects for assessments, *F*(6,318) = 9.58, *MS*_e_ = 0.239, *p* < 0.001, and serial positions, *F*(2,106) = 10.36, *MS*_e_ = 0.053, *p* < 0.001, and a non-significant Assessment × Serial Position interaction, *F*(12,636) = 1.37, *MS*_e_ = 0.016, *p* = 0.175. Bonferroni *post hoc* comparisons revealed significant age-related increases in rehearsal behavior between assessments one and two to seven, and between assessments two and five to six. In addition, there was an age-invariant recency effect for the last serial position compared to the first two serial positions (all *p*s < 0.05, Bonferroni-corrected).

The two-way repeated measures ANOVA with assessments (1–7) and serial positions (1–5) as within-subject factors revealed significant main effects for assessments, *F*(6,318) = 12.10, *MS*_e_ = 0.207, *p* < 0.001, and serial positions, *F*(4,212) = 27.17, *MS*_e_ = 0.091, *p* < 0.001, and a non-significant Assessment × Serial Position interaction, *F*(12,636) = 1.02, *MS*_e_ = 0.030, *p* = 0.434. Bonferroni *post hoc* comparisons revealed significant age-related increases in rehearsal behavior between assessments one and two to seven, between assessments two and five to six, and between assessments four and six, respectively. In addition, there were an age-invariant primacy effect and an extended recency effect: the first item was rehearsed with a higher probability than items from serial position 2 and 3 in this rehearsal set, the penultimate item was rehearsed with a higher probability than items from serial position 3, and the last item was rehearsed with a higher probability than all previously presented items (all *p*s < 0.05, Bonferroni-corrected).

The two-way repeated measures ANOVA with assessments (1–7) and serial positions (1–7) as within-subject factors revealed significant main effects for assessments, *F*(6,318) = 13.92, *MS*_e_ = 0.174, *p* < 0.001, and serial positions, *F*(6,318) = 14.65, *MS*_e_ = 0.084, *p* < 0.001, and a non-significant Assessment × Serial Position interaction, *F*(36,1908) = 0.76, *MS*_e_ = 0.030, *p* = 0.854. Bonferroni *post hoc* comparisons revealed that in this rehearsal set there was an age-related increase in rehearsal activity between assessment one and all subsequent assessments, between assessment two and assessments five to seven, and between assessment three and assessment six. In addition, rehearsal behavior in this rehearsal set was characterized by a primacy, an elevated middle-list effect, and a recency effect. Regarding primacy, comparisons revealed significant differences between serial positions 1 and 3–4, and 2 and 3–4. Elevated rehearsal for items from the middle was substantiated by significant comparisons between serial position 5 and 3–4. Regarding recency, comparisons between the last serial position and all previous serial positions, but the first one, turned out to be significant (all *p*s < 0.05, Bonferroni-corrected).

A two-way repeated measures ANOVA with assessments (1–7) and serial positions (1–9) as within-subject factors revealed significant main effects for assessments, *F*(6,318) = 10.78, *MS*_e_ = 0.136, *p* < 0.001, and serial positions, *F*(8,424) = 15.77, *MS*_e_ = 0.081, *p* < 0.001, as well as a non-significant Assessment × Serial Position interaction, *F*(48,2544) = 1.07, *MS*_e_ = 0.028, *p* = 0.343. Bonferroni *post hoc* comparisons revealed age-related increases between assessments one and two to seven, and between assessment two and assessments five to seven. Again, there was a primacy effect, including the first two items, a tendency for a middle-list effect, and a recency effect. Comparisons revealed higher rehearsal probabilities for the first serial position compared to all other but the last two serial positions, and significant higher rehearsal probabilities for serial position 2 compared to serial positions 4 and 7. Rehearsal probability turned out to be tendentially larger at serial position 5 compared to serial positions 4 (*p* = 0.062, Bonferroni-corrected) and 7 (*p* = 0.068, Bonferroni-corrected). A recency effect was demonstrated by significant differences between serial position 9 and all previous but the first serial position, and by a significant difference between serial position 8 and 7 (all *p*s < 0.05, Bonferroni-corrected).

#### Probability of First Recall

As stated above, PFR reveals the children’s initial response. Thus far, PFR had primarily been analyzed in the context of the recall phase. Under the assumption that rehearsal sets constitute a specific kind of recall during study, the final analyses examine PFR for four points in time during study, that is, for rehearsal sets 4, 6, 8, and 10. Similar to the previous analyses, the item that defined simultaneously the end of the precedent and the beginning of the current rehearsal set was excluded from the examinations. The reason behind this exclusion was to distinguish recall of items from the precedent rehearsal set and repetition of the currently presented item, respectively. Consequently, analyses of rehearsal set 4 included items from serial positions 1–3, analyses of rehearsal set 6 included items from serial positions 1–5, and so on. Previous analyses of PFR in adults had demonstrated that with increasing list length PFR changes from high values for the primacy item to elevated values for recency items (e.g., Grenfell-Essam and Ward, 2012). Accordingly, analyses in this section focus on items from the first and last serial position of the list thus far presented.

**FIGURE 3:**
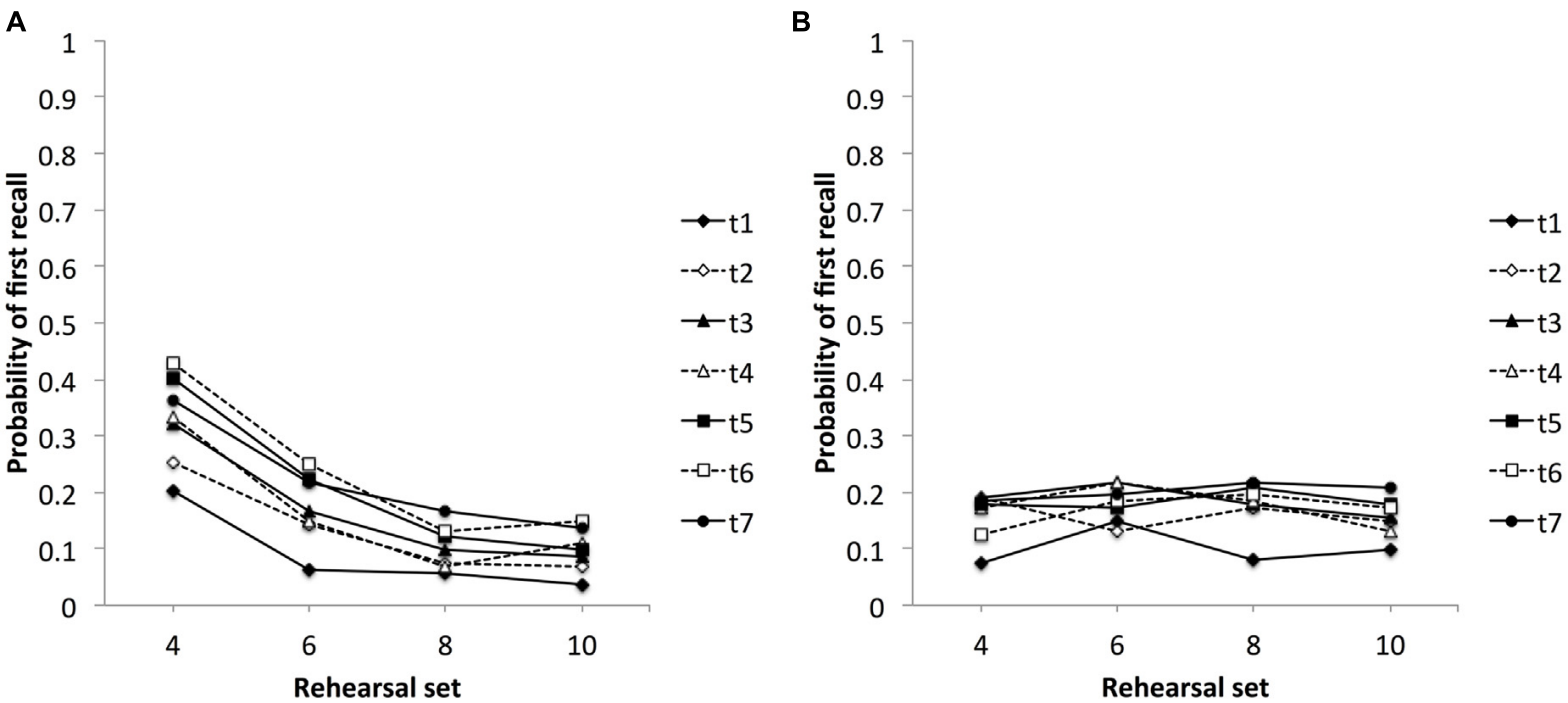
**Probability of first recall data as a function of rehearsal sets 4, 6, 8, and 10, for assessments (t) 1–7, respectively: for items from the first serial position (A), for items from the last serial position of the respective rehearsal set (B)**.

**Figures 3A,B** show the probabilities of first recall for rehearsal sets 4, 6, 8, and 10, for the first item from the list and the last item from the list section until then presented. Whereas the first item remained the same throughout all rehearsal sets, the last item was item from position *n*-1 of the respective rehearsal set. Four 7 (assessments) × 3 (serial positions 1, 2 to *n*-1, and *n*, for the respective rehearsal sets) repeated ANOVAs were performed on the PFR. For rehearsal set 4 the two-way repeated measures ANOVA with assessments (1–7) and serial positions (1, 2, 3) as within-subject factors revealed significant main effects for assessments, *F*(6,318) = 8.31, *MS*_e_ = 0.029, *p* < 0.001, and serial positions, *F*(2,106) = 33.19, *MS*_e_ = 0.194, *p* < 0.001, and a non-significant Assessment × Serial Position interaction, *F*(12,636) = 1.70, *MS*_e_ = 0.061, *p* = 0.063. Bonferroni *post hoc* comparisons revealed higher PFRs for assessments two to seven compared to assessment one. Independent of age, there was a strong primacy and a weaker recency effect. There was a significant tendency to start rehearsal in this rehearsal set with the first list item compared to items from the second and third serial position. Initial response differed, however, also between the latter positions with initial items coming more frequently from serial position 3 compared to serial position 2 (all *p*s < 0.05, Bonferroni-corrected). For rehearsal set 6 the two-way repeated measures ANOVA with assessments (1–7) and serial positions (1, 2–4, 5) as within-subject factors revealed significant main effects for assessments, *F*(6,318) = 6.13, *MS*_e_ = 0.027, *p* < 0.001, and serial positions, *F*(2,106) = 13.34, *MS*_e_ = 0.145, *p* < 0.001, and a non-significant Assessment × Serial Position interaction, *F*(12,636) = 1.55, *MS*_e_ = 0.044, *p* = 0.103. Bonferroni *post hoc* comparisons revealed higher PFRs for assessments three, five, six, and seven compared to assessment one and for assessment six compared to assessment two. Independent of age, there were strong primacy and recency effects (first and last serial position) compared to items from the middle of the thus far presented list (all *p*s < 0.05, Bonferroni-corrected). For rehearsal set 8 the two-way repeated measures ANOVA with assessments (1–7) and serial positions (1, 2–6, 7) as within-subject factors revealed significant main effects for assessments, *F*(6,318) = 6.66, *MS*_e_ = 0.021, *p* < 0.001, and serial positions, *F*(2,106) = 21.30, *MS*_e_ = 0.091, *p* < 0.001, and a non-significant Assessment × Serial Position interaction, *F*(12,636) = 0.99, *MS*_e_ = 0.044, *p* = 0.461. Bonferroni *post hoc* comparisons revealed higher PFRs for assessments three, five, six, and seven compared to assessment one. Independent of age, there was a strong primacy and an even stronger recency effect. That is, the tendency to start rehearsal in this rehearsal set was strongest for items from the last serial position (here, serial position 7) compared to all other serial positions. Initial rehearsal was, however, stronger for items from the first serial position compared to items from the middle of the thus far presented list (all *p*s < 0.05, Bonferroni-corrected). Finally, for rehearsal set 10 the two-way repeated measures ANOVA with assessments (1–7) and serial positions (1, 2–8, 9) as within-subject factors revealed significant main effects for assessments, *F*(6,318) = 4.26, *MS*_e_ = 0.025, *p* < 0.001, and serial positions, *F*(2,106) = 23.24, *MS*_e_ = 0.071, *p* < 0.001, and a non-significant Assessment × Serial Position interaction, *F*(12,636) = 0.68, *MS*_e_ = 0.033, *p* = 0.774. Bonferroni *post hoc* comparisons revealed higher PFRs for assessment five, six, and seven compared to assessment one. In addition, there was again a strong primacy and an even stronger recency effect. PFR was highest for items from the last serial position (here, serial position 9) compared to all other serial positions (note: serial position 9 compared to serial position 1: *p* = 0.05). Furthermore, items from the first serial position showed higher PFRs than items from the middle list positions. In sum, there were strong tendencies to start rehearsal in the respective rehearsal sets with items from the first and the last serial position. There was, however, a change with increasing list length: PFR was highest for the primacy item in the early section of the list and was highest for the recency item in the later sections of the list.

## Discussion

It has been demonstrated over and over again that active rehearsal of several items together is a critical determinant of recall performance in children. Likewise, many studies highlighted the developmental changes from passive rehearsal in younger children to active rehearsal in older children. The question, however, why this active rehearsal process in older children should facilitate recall performance remained mostly unexplored. In the present analyses this question was approached by starting from the end product of children’s learning process in free recall, namely, the serial position curve. Because the serial position curve is (only) the result of previous rehearsal behavior the current analyses additionally examined linkages between study and recall. To identify how rehearsal behavior and its recall consequences in free recall unfold, the rehearsal process was decomposed into single rehearsal events. The basic idea behind these analyses was that both rehearsals and recalls appear to consist of sequences of previously presented items and rehearsals of these items (Laming, 2006, 2008). Consequently, at each rehearsal opportunity the compilation of rehearsal sets and the resulting rehearsal patterns were assumed to be analogous to recall patterns. Accordingly, the free recall performance should be the end product of a cumulative iterative recall process.

In the present longitudinal study, recall performance increased with age. Further, serial position analyses clearly indicated age-related changes in the prerecency sections of the curves. When recall was plotted according to nominal serial positions, there was virtually no primacy effect at the first assessment while it increased with increasing age. According to the traditional view of developing rehearsal-recall linkages (e.g., Cuvo, 1975; Ornstein and Naus, 1978), the increasing primacy effect should reflect the consequences of an active rehearsal strategy including the first items in the list. Active rehearsal should result in the selective transfer of these early items into a LTS. Active rehearsal is thought to result in more varied interitem connections than passive rehearsal. Accordingly, items that are actively rehearsed should have stronger linkages to other items in LTS than do passively rehearsed items. Recency is, from this perspective, the consequence of emptying a STS first. Items in this STS are assumed to be recently added and therefore to come from the end of the list. Accordingly, STS influences seem to be independent of children’s age. The interpretation of distinct attributions of primacy and recency to LTS and STS, respectively, is challenged by the PFR serial position curves in the current study. These curves showed elevated probabilities for items from the end and from the beginning of the list to be recalled first. Whereas the effect for items from the end of the list proved to be age-invariant, the effect for items from the first list position turned out to increase with age. This latter increase suggests that increasing selective rehearsal behavior contributed to the tendency to start free recall with the first item.

This is supported by analyses in adults by Grenfell-Essam et al. (2013) who examined PFR under conditions when rehearsal was prevented (which is comparable to passive rehearsal behavior in younger children) and under conditions of (active) rehearsal behavior facilitated by slow presentation rates. Under the impeded rehearsal condition, participants demonstrated a reduction in the proportion of trials in which they initiated recall with the first item and under the (active) rehearsal condition participants demonstrated an elevated tendency to initiate recall with the first item. In addition to the discussed primacy and recency effects, serial position curves revealed elevated recall probabilities for list positions 5 and 8 in the middle portion of the list. The occurrence of those peaks proves the interpretation of distinct attributions of primacy and recency to LTS and STS, respectively, less straightforward. It rather points to rehearsal as a mechanism that assembles and organizes to-be-learned material into several groups of items. This interpretation is reviewed in more detail below.

Considering the findings on PFR, it seems obvious that rehearsal behavior is a key mechanism in ordering and reordering material from a list. Taking into account rehearsal behavior and linking this behavior to free recall, allows for a better understanding of study-recall processes. Corresponding to similar analyses previously done by Tan and Ward (2000) and Ward (2002) in adults, recall was replotted in terms of the functional ordering of the items. This reformulation of children’s recall data produced an elevated recency effect but no, or only little, primacy effect. Therefore, the observed primacy effect in children’s nominal serial position curves seems to occur because the items presented early in the list tended to be rehearsed in later rehearsal sets. Whereas the recency effect, obtained by replotting children’s recall data, was age invariant, recall of items from the prerecency portion of the list was age-related. These age-related differences may, however, essentially be ascribed to the general age-related increase in recall performance. In addition to the recency of rehearsals, also the number of rehearsals (Rundus, 1971; Tan and Ward, 2000) seems to play a crucial role for children’s free recall. In line with previous findings (see, e.g., Ornstein et al., 1975), items from the beginning of the list received much more rehearsals in newly compiled rehearsal sets than items from later list portions. There was a clear age-related increase in the amount of redintegrations in later rehearsal sets. Together with the functional serial position curves, the number of additional rehearsals appear to be good predictors for developmental changes in free recall on the basis of children’s rehearsal behavior. These two measures, however, seem to be particularly intertwined. The initial repetition of early list items may lead to the repetition of a rehearsal sequence which leads in turn to a perpetuated recency of this sequence. Due to this recency, accessibility of this sequence is increased and consequently further repetition is facilitated. In the present study, items from the first five serial positions were potentially more often repeated than items from later list portions and were accordingly more likely rehearsed toward the end of the list, that is, in later rehearsal sets (as will be discussed below). Consequently, the frequent repetition of a sequence and the recency of that sequence seem to be inseparable.

In developing a predictive algorithm, Laming(2006, 2008) demonstrated in adults that there is a strong relationship between previous sequences of items, rehearsals of these items, and free recall. During presentation of the list, items and rehearsals of these items generate an internal sequence. Each newly presented or rehearsed item is added or, respectively, readded to the head of this sequence. Consequently, Laming observed that rehearsals consisted of instantiations of runs and re-runs of previously presented and rehearsed items and suggested that these sequences are stored in memory. Accordingly, rehearsals and recalls should both be based on previous mnemonic records resulting in a cumulative mnemonic record. It follows that both should share some common memory mechanisms.

In the present study, children’s rehearsal behavior was decomposed into various rehearsal opportunities (rehearsal sets). Tracing the rehearsal process throughout the list should give some information about how rehearsal unfolds and how later patterns of rehearsal are based on previous patterns. In line with findings by Tan and Ward (2000) and Grenfell-Essam et al. (2013) in adults, the proportion of rehearsals as a function of serial position in the various rehearsal sets was similar to recall of lists of words with different list lengths. Independent of children’s age, all analyzed rehearsal sets (but the first rehearsal set) showed a one- or two- item recency effect. In addition, the early rehearsal sets were characterized by a one-item primacy effect whereas the later rehearsal sets showed a two-item primacy effect. Finally, a peak at serial position 5 also marked later serial position rehearsal patterns. This finding is particularly interesting because this peak clearly resembles the nominal serial position curves during recall. The stability of this peak throughout the study phase and recall phase suggests that recall was built on rehearsal patterns established through repeated rehearsal. Accordingly, children’s free recall seems to be cumulative because later patterns of rehearsal are related to previous patterns of rehearsal and the pattern of recall is related to rehearsal patterns in the study phase.

The assumption that rehearsal at each rehearsal opportunity equates to some extent to recall of the thus far presented material (see also, Ward et al., 2010) was additionally supported by analyses of PFR in various rehearsal sets. At rehearsal opportunities early in the list (few thus far presented items) the tendency to initiate rehearsal with the first list item was larger than the respective tendency to start with the lastly presented item. In the course of the list and therefore with increasing number of to-be-memorized items, PFR shifted to a tendency to initiate rehearsal with the lastly presented item (note that lastly presented item refers to the item that initiated the last interstimulus interval before PFR was examined; as such, PFR examined rehearsal of previously presented items but not mere repetition of the currently presented item). These findings have to be evaluated with a certain degree of caution because at later rehearsal opportunities there were age-related rehearsal differences: children demonstrated more active rehearsal behavior (with larger rehearsal sets) when they were older than when they were younger (for more detailed analyses, see Lehmann and Hasselhorn, 2007). However, due to the lack of any age-related interaction in the analyses, these findings seem to be in line with findings from a set of experiments with adult participants by Grenfell-Essam et al. (2013) who analyzed rehearsal and respective recall consequences under conditions of different list lengths and rehearsal instructions. They demonstrated that participants mainly initiated recall with the first item from the list on short lists and that participants mainly initiated recall with one of the last four items on longer lists, respectively. They concluded, however, that rehearsal was not the sole explanation for initial recall coming from the first serial position when lists were short. Under the condition in which rehearsal was disrupted by articulatory suppression, participants still tended to initiate recall with the first list item, albeit to a lesser degree. The latter finding matches well the findings from the present study. When children were younger they used active rehearsal behavior to a lesser extent. Nevertheless, they tended to initiate rehearsal behavior within rehearsal sets with the first list item, especially early in the list. With increasing list length, age-related differences increased and children were less likely to start rehearsal with the first list item when they were younger compared to when they were older. Most evident was the age-related, and therefore supposedly rehearsal based difference, in initiating recall with the first list item. Accordingly, under conditions of slow presentation rate, as was the case in the present study, rehearsal seems to contribute largely to this tendency in children when they are older. The observation that rather passively rehearsing younger children initiated rehearsal at early rehearsal opportunities with the first list item may point to the involvement of a primacy gradient with the first list item given more encoding than subsequently presented items. This assumption will be discussed briefly below.

The analyses in adults by Laming (2006, 2008) with unstructured material and early studies on rehearsal behavior in children by Ornstein et al. (1975) with semantically organized and randomly organized material suggest that structure in the runs of rehearsal of previously presented items is not the consequence of mere chance but that rehearsals and sequences of rehearsals follow certain regularities. Under the condition of item visibility after their presentation, Ornstein et al. (1975) found that children demonstrated less rehearsal behavior, when the list organization was very explicit but demonstrated elevated rehearsal behavior when list organization was less obvious. They concluded that under random organization of the material, rehearsal behavior took the place of semantic organization and was therefore the important factor for remembering the randomly structured items. Accordingly, rehearsal should serve an organizational function. In the present study with semantically unrelated material, almost all analyses indicate that rehearsal may serve as a grouping mechanism. The nominal serial position curves in rehearsal sets 8 and 10 and both nominal and functional serial position curves during recall exhibit striking peaks at serial position 5 and 8, respectively. In addition the PFR during recall and the number of additional redintegrations in newly compiled rehearsal sets show again peaks at serial position 5 and to a lesser extent at serial position 8. These peaks are usually known to occur if the to-be-learned material is semantically related and therefore organizable into categories (e.g., Ornstein et al., 1975) or temporally grouped (e.g., Hitch et al., 1996).

Under the assumption that children’s rehearsal behavior served as a grouping mechanism, the findings from the current study may well be explained by a recently proposed model of short-term memory and episodic memory by Farrell (2012). In this model, people are assumed to group several items from a list into episodic clusters according to their temporal proximity. The items are associated with a group context and with information about their position within the group. The strength of encoding of items within groups is driven by a primacy gradient during input. Hence, participants group items into clusters and within each group, items are qualified by their position within the group. The latter principle is assumed to function according to the temporal order of presentation of the items. During recall, a group context for the group has to be retrieved. Once the group context is retrieved, it is used together with a cue for within-group positioning of the item. Here lies the strongest cause for forgetting: a cluster cannot be accessed if the cluster’s context cannot be retrieved. However, if the group context has been carried over from the list presentation, as is the case in the last cluster of a list, the group context does not have to be retrieved and this group can be recalled immediately, accounting for the recency effect. The primacy effect in this model arises from several mechanisms: the group context of the first group is specific because it has only subsequent but no prior neighboring groups; the primacy gradient of within group items additionally emphasizes the first items of the list.

In the following, I will elaborate a scenario characterizing children’s rehearsal behavior in the present study that may result from the mechanisms of Farrell’s (2012) model and the assumption that rehearsals and recalls consist of the presentations and rehearsals of previously presented items: Early in the list, children include items according to their temporal proximity (note that Lehmann and Hasselhorn, 2012 found that rehearsal sequences in children consisted mainly of items in their original temporal order and that this effect was largest early in the list). Up to a certain number of items and due to respective active rehearsal of these items, the group context is continuously carried over to the next rehearsal opportunity. An elevated PFR for the first list item should be the consequence of the primacy gradient within the list. Early in the list, there may be only one group. Consequently, the respective group context does not have to be actively retrieved and even younger children reveal elevated PFR for the first list item due to the primacy gradient and due to an edge effect of the first group’s first item. Because organization was not salient in the present study, group size may have been determined by the children. Accordingly, group size of the first group seems to land at five items, independent of children’s age. It seems that after presentation of the items at serial position 5, a new group context is established. Analyses of rehearsal sets 8 and 10 suggest that children are repeatedly able to adequately retrieve the first group’s context and in rehearsal set 8 also the second group’s context. As item presentation continues, however, they seem to fail to retrieve the latter context and therefore demonstrate reduced recall from this group. Finally, children’s elevated recall of the recency portion of the list may again be ascribed to a carried over representation of this last group’s context. The age-related elevated PFR for the first list item during recall suggests that children better retrieve the first group’s context when they are older compared to when they are younger. The reason may be based on a carried over context due to recent rehearsal of items from the first group (supported by the elevated number of additional rehearsals for early list items). Another interpretation is that by carrying the group across each rehearsal opportunity (indicated by similar nominal serial position patterns), older children form a hierarchical structure of the sequence of items (similar to multitrial free recall), which in turn facilitates retrieval of the first group’s context (Farrell, 2012).

The execution of an active rehearsal strategy is an effortful mental process that consumes the child’s limited mental resources (Guttentag et al., 1987). Age-related differences in the execution of active rehearsal may be based on how efficiently it can be carried out. The high processing demands of active rehearsal seem to restrict the usage of this strategy in younger children. In older children, the execution of cumulative rehearsal may free mental resources that can be used to augment the execution of the strategy with other components.

Accordingly, individual differences and developmental changes in available mental resources may account to a large extent for the variability in successful use of active rehearsal (see also, Lehmann and Hasselhorn, 2007). For instance, older children and children with higher working memory capacity should be able to assemble early presented items into larger subsequences. In addition, they should effectively rehearse these items to the end of the list, leading to superior recall. In contrast, younger children and children with low working memory capacity, should, if at all, compile smaller early subsequences which additionally may be less stable (regarding content and order). Accordingly, early list items should end up further from the end of the list, leading to inferior recall of primacy and prerecency items.

The detailed analyses presented here, illustrate the complexities of rehearsal-recall processes in children. There are, however, a number of underlying processes beyond memory capacity that must be accounted for to capture the complete picture of children’s rehearsal-recall development. Future investigations may specify these underlying developmental processes. Consider, for example, the importance of children’s metamemory (e.g., DeMarie et al., 2004) for the production and efficiency of active rehearsal behavior. Future research may address the question of whether high memory capacity suffices to produce active rehearsal when presented with a list of words or of whether metamemory may serve a mediating role. Regarding rehearsal processes throughout the list, future research may address the question of whether grouping of the items into subsequences is based on an organizational plan that is consciously developed by the children. In this regard, children’s metacognitive knowledge about their own capacity limits or about how to deal with these limits may play a crucial role. I hope that the findings presented here highlight the need for further fine-grained analyses and for the identification of underlying mechanisms to gain a deeper understanding of the relationship between rehearsal and recall processes.

In summary, this study provided evidence that in children the mechanisms that produce patterns of recall seem to be the same that produce patterns of rehearsal. Accordingly, developmental changes in the patterns of recall were accompanied by similar changes in patterns of rehearsal and vice versa. I have presented two sets of analyses that focused on the rehearsal-recall relationship from two different perspectives. First, age-related differences in recall patterns were interpreted as a result of selective rehearsal behavior that changed with age. When children were older, items were rehearsed more often and were rehearsed further toward the end of the list. This behavior resulted in higher recall probabilities for items that were close to the point of recall. Second, analysis of several rehearsal sets during study revealed that rehearsal behavior at each rehearsal opportunity seemed to be based on previous rehearsal behavior. With increasing age, children were more and more able to redintegrate previously presented and rehearsed items into subsequent rehearsal sets. Accordingly, patterns of rehearsal of previous rehearsal sets echoed in newly compiled rehearsal sets. This behavior persisted throughout the whole rehearsal process, that is, it was apparent at each rehearsal opportunity, and it was ultimately evident during recall.

## Conflict of Interest Statement

The Guest Associate Editor, Christopher Jarrold, declares that, despite having collaborated with author, Martin Lehmann, the review process was handled objectively and no conflict of interest exists. The author declares that the research was conducted in the absence of any commercial or financial relationships that could be construed as a potential conflict of interest.

## References

[B1] Atkinson R. C. Shiffrin R. M. (1968). “Human memory: a proposed system and its control processes,” in *The Psychology of Learning and Motivation* Vol. 2 eds Spence K. W. Spence J. T. (New York, NY: Academic Press).

[B2] Cuvo A. J. (1975). Developmental differences in rehearsal and free recall. *J. Exp. Child Psychol.* 19 265–278 10.1016/0022-0965(75)90090-9

[B3] DeMarie D. Miller P. Ferron J. Cunningham W. (2004). Path analysis tests of theoretical models of children’s memory performance. *J. Cogn. Dev.* 5 461–492 10.1207/s15327647jcd0504_4

[B4] Farrell S. (2012). Temporal clustering and sequencing in short-term memory and episodic memory. *Psychol. Rev.* 119 223–271 10.1037/a0027371 22506678

[B5] Grenfell-Essam R. Ward G. (2012). Examining the relationship between free recall and immediate serial recall: the role of list length, strategy use, and test expectancy. *J. Mem. Lang.* 67 106–148 10.1016/j.jml.2012.04.004

[B6] Grenfell-Essam R. Ward G. Tan L. (2013). The Role of rehearsal on the output order of immediate free recall of short and long lists. *J. Exp. Psychol. Learn. Mem. Cogn.* 39 317–347 10.1037/a0028974 22774850

[B7] Guttentag R. E. Ornstein P. A. Siemens L. (1987). Children’s spontaneous rehearsal: transitions in strategy acquisition. *Cogn. Dev.* 2 307–326 10.1016/S0885-2014(87)80010-2

[B8] Hitch G. J. Burgess N. Towse J. N. Culpin V. (1996). Temporal grouping effects in immediate recall: A working memory analysis. *Q. J. Exp. Psychol. Hum. Exp. Psychol.* 49 116–139 10.1080/027249896392829

[B9] Laming D. (2006). Predicting free recalls. *J. Exp. Psychol. Learn. Mem. Cogn.* 32 1146–1163 10.1037/0278-7393.32.5.1146 16938052

[B10] Laming D. (2008). An improved algorithm for predicting free recalls. *Cognit. Psychol.* 57 179–219 10.1016/j.cogpsych.2008.01.001 18329010

[B11] Laming D. (2010). Serial position curves in free recall. *Psychol. Rev.* 117 93–133 10.1037/a0017839 20063965

[B12] Lehmann M. Hasselhorn M. (2007). Variable memory strategy use in children’s adaptive intratask learning behavior: developmental changes and working memory influences in free recall. *Child Dev.* 78 1068–1082 10.1111/j.1467-8624.2007.01053.x 17650126

[B13] Lehmann M. Hasselhorn M. (2010). The dynamics of free recall and their relationship to rehearsal between 8-and 10-years of age. *Child Dev.* 81 1006–1020 10.1111/j.1467-8624.2010.01448.x 20573119

[B14] Lehmann M. Hasselhorn M. (2012). Rehearsal dynamics in elementary school children. *J. Exp. Child Psychol.* 111 552–560 10.1016/j.jecp.2011.10.013 22196371

[B15] Ornstein P. A. Medlin R. G. Stone B. P. Naus M. J. (1985). Retrieving for rehearsal: an analysis of active rehearsal in children’s memory. *Dev. Psychol.* 21 633–641 10.1037/0012-1649.21.4.633

[B16] Ornstein P. A. Naus M. J. (1978). “Rehearsal Processes in children’s memory,” in *Memory Development in Children* ed. Ornstein P. A. (Hillsdale, NJ: Erlbaum) 69–99.

[B17] Ornstein P. A. Naus M. J. Liberty C. (1975). Rehearsal and organizational processes in children’s memory. *Child Dev.* 46 818–830 10.2307/1128385

[B18] Ornstein P. A. Naus M. J. Stone B. P. (1977). Rehearsal training and development differences in memory. *Dev. Psychol.* 13 15–24 10.1037/0012-1649.13.1.15

[B19] Raaijmakers J. G. W. Shiffrin R. M. (1981). Search of associative memory. *Psychol. Rev.* 88 93–134 10.1037/0033-295X.88.2.93

[B20] Rundus D. (1971). Analysis of rehearsal processes in free recall. *J. Exp. Psychol.* 89 63–77 10.1037/h0031185

[B21] Rundus D. Atkinson R. C. (1970). Rehearsal processes in free recall: a procedure for direct observation. *J. Verbal Learn. Verbal Behav.* 9 99–105 10.1016/S0022-5371(70)80015-9

[B22] Tan L. Ward G. (2000). A recency-based account of the primacy effect in free recall. *J. Exp. Psychol. Learn. Mem. Cogn.* 26 1589–1625 10.1037/0278-7393.26.6.1589 11185785

[B23] Ward G. (2002). A recency-based account of the list length effect in free recall. *Mem. Cognit.* 30 885–892 10.3758/BF03195774 12450092

[B24] Ward G. Tan L. (2004). The effect of the length of to-be-remembered lists and intervening lists on free recall: a re-examination using overt rehearsal. *J. Exp. Psychol. Learn. Mem. Cogn.* 30 1196–1210 10.1037/0278-7393.30.6.1196 15521798

[B25] Ward G. Tan L. Grenfell-Essam R. (2010). Examining the relationship between free recall and immediate serial recall: the effects of list length and output order. *J. Exp. Psychol. Learn. Mem. Cogn.* 36 1207–1241 10.1037/a0020122 20804293

